# The psychological impact on and mental health outcomes for victim-survivors of technology-assisted child sexual abuse: a systematic literature review

**DOI:** 10.3389/fpsyg.2026.1682155

**Published:** 2026-04-10

**Authors:** Alice Forrester Fellowes, Juliane A. Kloess, Sarah Gladden

**Affiliations:** 1 School of Health in Social Science, University of Edinburgh, Edinburgh, United Kingdom; 2 The Orchard Clinic, Royal Edinburgh Hospital, Edinburgh, United Kingdom

**Keywords:** child sexual abuse, online child sexual exploitation, online safety, technology, trauma, victimization

## Abstract

**Background:**

The increased use of online technologies has expanded the social landscape for individuals with ill-intent to sexually exploit and abuse children. To date, the specific psychological impact on and mental health outcomes for children who have experienced Technology-Assisted Child Sexual Abuse (TA-CSA) remain underexplored in comparison to those of CSA in the physical world.

**Aim:**

This mixed-methods systematic literature review synthesizes the findings from existing studies that have explored these phenomena.

**Method:**

A systematic search of three databases (EMBASE, MEDLINE, and PsycINFO) was conducted. Included articles (*n* = 18) were assessed for quality using the Mixed Methods Appraisal Tool. The findings were integrated using a narrative thematic approach.

**Results:**

The synthesis yielded seven key themes: (i) An Onslaught of Painful Emotions, (ii) A Shattered Sense of Self: Self-Blame and Guilt, (iii) A Future Held Hostage: Image Permanence and Hypervigilance, (iv) Broken Bonds: Social Isolation and Relational Rupture, (v) Prevalence of Mental Health Difficulties, (vi) Behavioral and Interpersonal Sequelae, and (vii) Gender-Based Variations in Psychological Outcomes.

**Conclusions:**

The findings reveal that experiences of TA-CSA lead to a range of significant mental health outcomes, from profound emotional distress and a shattered sense of self to a high prevalence of psychological? Difficulties. The unique features of TA-CSA, such as digital permanence, are associated with persistent trauma responses. Implications for clinical practice, policy, and future research are discussed.

**Systematic review registration:**

PROSPERO registration number CRD42024548986.

## Introduction

Child sexual abuse (CSA) facilitated by online technologies is a growing global concern, amplified by the increasing immersion of youth in digital spaces. Recent studies suggested that the percentage of children under 18 years of age with their own phone and internet accessibility had risen to 84% in 2022 (Davidson et al., 2022; Rideout and Robb, 2019). With 60% of children reporting daily internet access, online technologies are now an integral part of their social and developmental lives (Jeney, 2015). Such rising trends are concerning, given that many children share personal information, as well as photos and videos, with individuals they are not acquainted with (Livingstone et al., 2017). According to Childlight (2024), CSA facilitated by online technologies affected 300 million children globally in 2024, with reported rates comparable, if not higher, than CSA that took place in the physical world.

While the sexual abuse of children has existed long before the emergence of the internet, its rapid development creates opportunities for individuals to harm children in new and less regulated spaces (Canadian Centre for Child Protection, 2017). Traditionally, CSA has been defined as

“*the involvement of a child in sexual activity that he or she does not fully comprehend, is unable to give informed consent to, or for which the child is not developmentally prepared and cannot give consent, resulting in actual or potential harm to the child's health, survival, development or dignity in the context of a relationship or responsibility, trust or power.”* (World Health Organization, 1999, p. 15)

Although this definition remains applicable, the inclusion of digital technologies has required a modification and expansion of existing definitions of CSA. Crucially, research highlights key differences between online and offline CSA, with the online environment facilitating distinct types of exploitation, and creating unique impacts that have not previously been documented (Hamilton-Giachritsis et al., 2021). This has resulted in the coining of term Technology-Assisted Child Sexual Abuse (TA-CSA) to refer to the sexual abuse of a child under the age of 18 years that has involved an online element (Hamilton-Giachritsis et al., 2020). Its additional complexities and features include, at the direction of offenders, the creation of non-consensual imagery, sexualised talk, engagement in or performance of often degrading sexual acts, including via webcam, stalking, and blackmail and/or threats to disseminate any of the material (Finkelhor et al., 2022; Joleby et al., 2020; Whittle et al., 2013).

The difficulty of clearly defining what constitutes TA-CSA is an ongoing debate within the wider academic literature (e.g., Kloess et al., 2019, 2021). Despite efforts to establish a standardized definition, the ever-evolving nature of technology and digital crimes make the categorization thereof a challenging task (Finkelhor et al., 2023). (Finkelhor et al. 2022) argues that it is important to recognize the distinct types of abuse experiences within TA-CSA, and how their respective harms are assessed, measured, and understood. While overarching terms, such as TA-CSA, offer inclusivity, different abuse experiences that involve technology carry with them varying implications for victim-survivors.

The impact of CSA on victim-survivors has long been established within the academic literature (Browne and Finkelhor, 1986). However, more recently, research has focused on the distinctive role the online environment plays within TA-CSA that may lead to differential and compounded psychological impacts on and mental health outcomes for victim-survivors (Hamilton-Giachritsis et al., 2020; Joleby et al., 2020), although this requires further in-depth exploration. For example, the dissemination of non-consensual imagery has been identified as a key feature of TA-CSA that will likely result in victim-survivors experiencing significant psychology distress and trauma responses, in light of the often violent, degrading and humiliating content of this material (Martin, 2014).

More specifically, awareness of such material existing or being in circulation has been reported to contribute to higher levels of post-traumatic stress disorder (PTSD) in victim-survivors, compared to abuse experiences that have not been “documented” (Joleby et al., 2020). The digital nature and existence of such material means that there is a permanent record, that it can be duplicated and stored an infinite number of times, and that it is accessible and can be retrieved within seconds across the world. Consequently, TA-CSA is not a one-off event, but a potentially lifelong threat of re-exposure, limiting opportunities for psychological healing, and directly contributing to higher levels of distress (Ali and Paash, 2022). (Insoll et al. 2022) further builds on these findings by highlighting the ongoing re-victimization of victim-survivors of TA-CSA every time material of their abuse experiences is disseminated and/or viewed. This leads to chronic states of hypervigilance, with victim-survivors reporting intense fear around material resurfacing, helplessness and powerlessness (Gewirtz-Meydan et al., 2019), as well as the potential to impact relationships and future educational and/or employment opportunities (Martin, 2014).

Another unique feature of TA-CSA is the insidious nature of grooming. Through manipulation of trust over time, boundaries are often blurred between exploitation and what may appear as a consensual relationship. This makes it challenging for victim-survivors to protect themselves, and carry with it a profound sense of guilt and shame for they believe that they participated in the abuse, and that it was their fault (Stänicke et al., 2024). While some studies attribute feelings of shame to victim-survivors developing an altered sense of self and how they view themselves (Mandau, 2021), others link them to delayed disclosures, often also underpinned by the sense of betrayal, all of which impact their ability to form healthy relationships in the future (Joleby et al., 2020).

Herman's (1992) theory of Complex PTSD (CPTSD) provides a critical framework for conceptualizing these unique and enduring impacts. Unlike traditional PTSD models derived from singular traumatic events, Herman argues that prolonged, repeated victimization characterized by a state of captivity, whether physical or psychological, results in a distinct and more pervasive spectrum of injury. In the context of TA-CSA, the digital permanence of abuse material functions as a psychological equivalent to this captivity, with victim-survivors being trapped in a cycle of continued contact with the perpetrator, and/or the abuse experience. This complex syndrome encompasses both symptomatic distress and profound characterological changes, including deformations of identity and relatedness, and a specific vulnerability to repeated harm and victimization. Herman's framework therefore offers an important theoretical lens for examining how the coercive dynamics of TA-CSA lead to multi-faceted and enduring harm.

Overall, this counters claims that experiences of TA-CSA do not have the same psychological impact on and mental health outcomes for victim-survivors of CSA in the physical world (e.g., Finch et al., 2020). In fact, (Finkelhor et al. 2023) suggests that TA-CSA, or abuse experiences that involve an online element, have the potential to cause a 50% greater impact, attributed to their compounding nature. More specifically, victim-survivors who have experienced TA-CSA are often subjected to multiple forms of victimization, including grooming, engagement in, and performance of sexual acts at the direction of offenders, and the production and dissemination of abuse imagery (Chauviré-Geib and Fegert, 2021). This is akin to poly-victimization, which has been reported to increase the likelihood of negative long-term effects when compared to a single abuse experience (Felitti et al., 2019), and is often exacerbated in cases of TA-CSA where multiple perpetrators are involved (Finkelhor et al., 2023).

The challenge in understanding the full extent of the psychological impact on and mental health outcomes for victim-survivors of TA-CSA is further underpinned by the low rates of disclosure and reporting of abuse experiences (Schmidt et al., 2023), and the fact that only those who are willing to share or report on their abuse experiences are represented in existing research. This is in part due to (i) the generally poor awareness and understanding of the nature and severity of TA-CSA (Darkness to Light, 2015), as well as uncertainty in victim-survivors around whether their experiences would count as abuse, especially in light of the absence of physical proximity (Quayle et al., 2012), and (ii) the known reality for and fear in victim-survivors of being blamed, shamed, and retaliated against (Martin and Slane, 2015; UNICEF, 2017). In addition, the perceived anonymity of the online environment may make the prospect of identifying an offender seem unlikely and futile (Ali and Paash, 2022).

It is therefore paramount to synthesize existing knowledge and understanding of the psychological impact of and mental health outcomes for victim-survivors of TA-CSA who have come forward and taken part in research, not only to attempt to provide a coherent overview, but also to highlight any gaps and inconsistencies within the existing evidence base. For example, (Page et al. 2025) suggests that the absence of standardized measures makes it difficult to compare findings across studies, and clearly determine the most frequently reported outcomes. Nevertheless, while many studies link TA-CSA to adverse outcomes, few systematically offer insights into why certain mental health outcomes arise, and how this may differ across victim gender and type of abuse experience, as well as be impacted by factors, such as the gender of and relationship to the offender (Chauviré-Geib and Fegert, 2021).

### The current review

The aim of the review presented here is to provide an overview of current knowledge and understanding of the psychological impact on and mental health outcomes for victim-survivors following experiences of TA-CSA, and thereby answer the following research question: (1) What are the psychological impacts on and mental health outcomes for victim-survivors who have experienced TA-CSA? To the authors' knowledge, this is the first mixed-methods review to synthesize findings from available studies of any design.

## Method

Prior to commencing the review, a preliminary scoping search was conducted across three databases (i.e., EMBASE, MEDLINE, PsycINFO). PROSPERO was searched to confirm that no prior review had been completed or was in progress on a research question similar to that of the present review.

### Search strategy

Given the absence of any existing systematic literature reviews in the area of interest, no time limits were placed on the search to maximize the identification of relevant studies. The SPIDER tool (sample, phenomenon of interest, design, evaluation and research type) was used to develop a search strategy (Cooke et al., 2012), and allow for the identification of qualitative, quantitative, and mixed-methods studies (Noyes et al., 2008). The search strategy was developed in consultation with a librarian. Table 1 provides an overview of search strategy according to the SPIDER tool.

**Table 1:** Search strategy using the SPIDER tool (Cooke et al., 2012).

SPIDER	Descriptors	Search terms
Sample	Children	Child^*^ or Youth^*^ or Boy^*^ or Girl^*^ Female^*^ or Male^*^ or Minor^*^ or Juvenile^*^ or “Young Pe^*^” or Adolescent^*^ or “Pre-adult^*^” or “Preschool” or Teen
Phenomenon of	Experienced abuse	Abuse or “Sexual Abuse^*^” or “Child Abuse” or “Sex^*^ Expl^*^” or Sexto^*^
Interest	Online	Online or “Sextort^*^” or “Digital” or Technol^*^ or “Cyber^*^” or “Online Sexual Expl^*^” or “Technology Assisted” or Internet or Grooming or “Online Sex^*^ Beh^*^” or “Online Sex^*^ Solicit^*^” or “Online Sex^*^ Exploit^*^”
Design	Any	N/A
Evaluation	Psychological impact	“Psychological Impact^*^” or “Psychological Consequence^*^” or “Psychological Effect^*^” or “Psychological Disturb^*^” or “Psychiatric Impact^*^” or “Psychological Disorder^*^” or “Psychiatric Consequence^*^” or “Psychiatric Effect^*^” or “Mental Health” or “Mental Illness” or “Mental Health Need^*^” or Trauma^*^ or Distres^*^ or Depress^*^ or PTSD or Shame^*^ or “Personality Disor^*^”
Research type	Any	N/A

^*^indicates search strategy table is a truncation. A technique used to broaden search results by finding multiple variations of a words ending.

### Search results

The search was conducted on 4th June 2024 and resulted in the return of 5,075 articles. One thousand six hundred eighty-eight duplicates were removed, resulting in a remaining total of 3,386. Six articles were identified following a citation search relating to the topic area, totaling 3,393 articles available for screening. To ensure methodological rigor, the search was restricted to peer-reviewed articles. Consequently, gray literature and unpublished reports were excluded. Using the inclusion criteria presented in Table 2, the researcher proceeded to screen titles and abstracts, excluding 3,325 articles.

**Table 2:** Inclusion and exclusion criteria.

Inclusion criteria	Exclusion criteria
i) Articles including participants aged up to 18 years, or adults (18+) reflecting retrospectively on victimization experience prior to the age of 18.	i) Articles focusing on child sexual abuse in the physical world (i.e., not involving an online element of the abuse experience).
ii) Articles exploring professionals or caregivers with direct experience of working with or supporting victim-survivors following TA-CSA.	ii) Articles focusing on offenders of child sexual abuse.
iii) Articles specifically exploring the psychological impact and/or mental health outcomes of victim-survivors following TA-CSA.	iii) Articles not specifically exploring the psychological impact and/or mental health outcomes of victim-survivors following TA-CSA.
iv) Peer-reviewed articles.	iv) Reviews, book chapters, gray literature, and policy reports.
v) Written in English language.	v) Written in a language other than English.
vi) Full-text available.	

**Figure 1:**
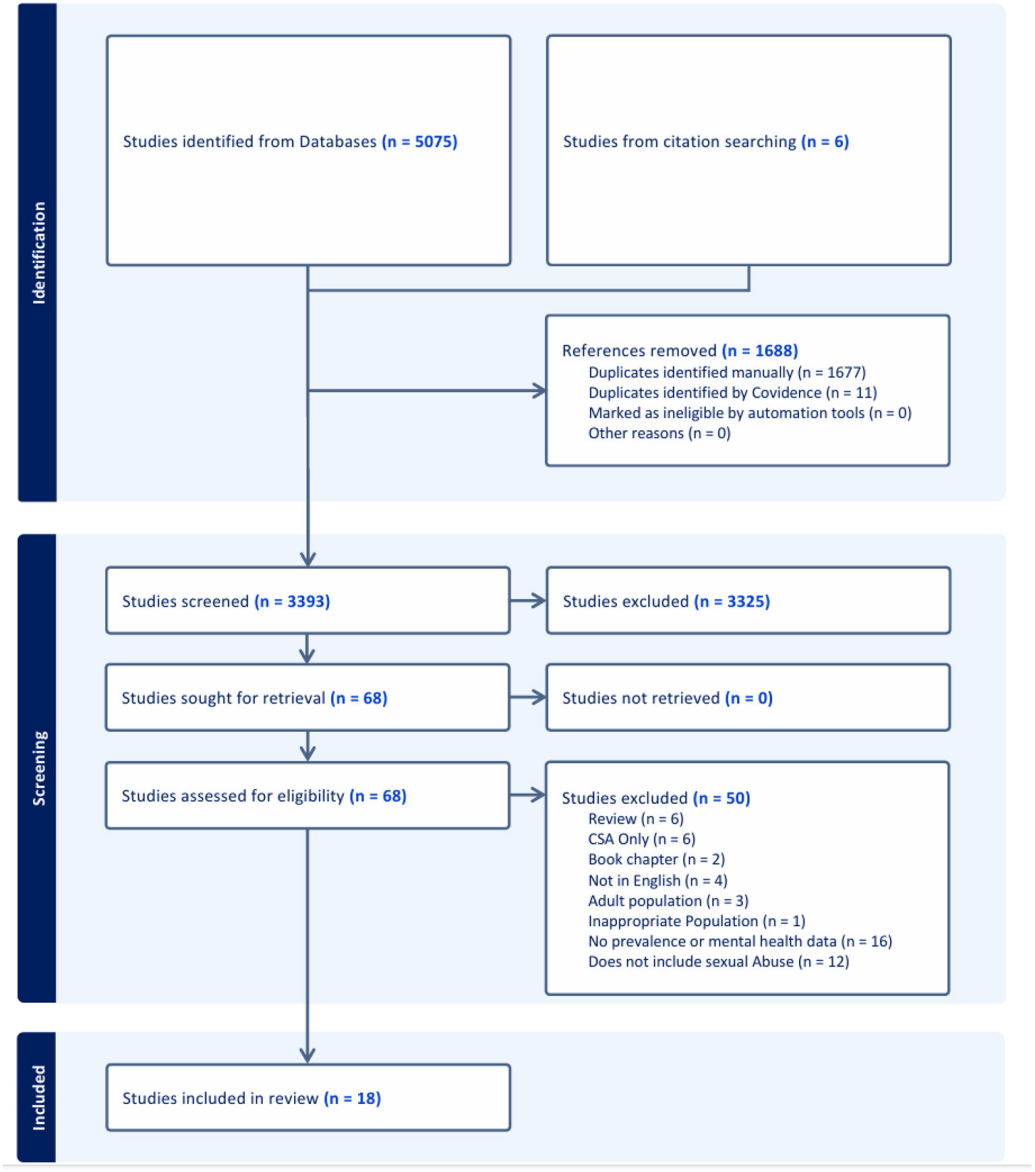
PRISMA (Page et al., 2020).

The researcher screened 68 articles in full, with 18 of these meeting the inclusion criteria. Of the final set of articles (*n* = *18*), nine had used a quantitative design, five had used a qualitative design, and four had used a mixed-methods design. Figure 1 presents the PRISMA flow diagram of the article selection process.

### Overview of studies

All studies included demographic information of their sample. Fourteen studies included only children as participants (De Santisteban and Gámez-Guadix, 2018; Dönmez and Soylu, 2019; ECPAT International, 2022; Gámez-Guadix et al., 2021; Gemara et al., 2022; Guerra et al., 2022; Guerra et al., 2021; Hamilton-Giachritsis et al., 2020; Joleby et al., 2021; Manrai et al., 2021; Martin, 2014; Say et al., 2015; Whittle et al., 2013; Ybarra et al., 2004). Two studies included only professionals working with children who had experienced TA-CSA (Martin, 2014; Wells and Mitchell, 2007). Two studies included both children and professionals within their sample (Hamilton-Giachritsis et al., 2017, 2021).

Eight of the quantitative studies were of a cross-sectional design, and analyzed data at a singular point in time (Dönmez and Soylu, 2019; Gámez-Guadix et al., 2021; Gemara et al., 2022; Guerra et al., 2021, 2022; Say et al., 2015; Wells and Mitchell, 2007; Ybarra et al., 2004). One study was of a repeated-measures design, and analyzed data across two time points (De Santisteban and Gámez-Guadix, 2018). The studies administered a variety of measures to assess the psychological impact of and mental health outcomes following TA-CSA.

More specifically, (Gámez-Guadix et al. 2018) used the Questionnaire of Sexual Solicitation and Interactions with Adults. Derogatis and Fitzpatrick's (2004) Brief Symptom Inventory (BSI) for Depression was also used by (De Santisteban and Gámez-Guadix 2018), and analyzed using structural equation modeling. The Strength and Difficulties Questionnaire by (Goodman 1997) and Pynoos et al.'s (1987) Child Post-Traumatic Stress Disorder Reaction Index were both deployed by (Dönmez and Soylu 2019), and analyzed using independent *t*-tests and Mann-Whitney U-tests. (Gámez-Guadix et al. 2021) applied their own Multidimensional Online Grooming Questionnaire, alongside their previously developed Questionnaire for Online Sexual Solicitation and Interactions with Adults (Gámez-Guadix et al., 2018), and the BSI (Derogatis and Fitzpatrick, 2004), undertaking exploratory and confirmatory factor analyses. The Multidimensional Scale of Perceived Social Support (Zimet et al., 1988), the Depression Self-Rating Scale (Birleson, 1981), and the Antisocial and Criminal Behavior Scale in Adolescents (Andreu and Peña, 2013), were used by (Guerra et al. 2022), and analyzed using regression analyses. In a previous study by (Guerra et al. 2021), the authors conducted a secondary analysis of the National Poly-Victimization Survey (Consejo Nacional De La Infancia, 2018), using ANOVA to analyze the data.

The DSM-IV (American Psychiatric Association, 2000) was used by (Say et al. 2015), alongside the Denver Developmental Screening Test (Frankenburg and Dodds, 1967), the WISC-R (Wechsler, 1974), and the WAIS-R (Wechsler, 1981), to conduct a multivariate regression analysis. (Wells and Mitchell 2007) utilized the DSM-IV (American Psychiatric Association, 2000) in combination with The Survey of Mental Health Issues and Inventory of Problematic Internet Experiences (Mitchell et al., 2005), conducting bivariate analyses. (Mitchell et al. 2001) developed the Youth Internet Safety Survey, and used logical regression to analyze the data. (Ybarra et al. 2004) further developed this research by using secondary data from the Youth Internet Safety Survey (Mitchell et al., 2001), and employing the DSM-IV (American Psychiatric Association, 2000) to investigate depressive symptomology using logistic regression.

Three qualitative studies used the method of semi-structured interviews (Gemara et al., 2022; Martin, 2014; Whittle et al., 2013; ECPAT International, 2022 utilized unstructured interviews, and (Manrai et al. 2021) used a combination of both interviews and focus groups. Of the mixed-methods studies, (Hamilton-Giachritsis et al. 2020) used interviews alongside a series of measures, including the Depression Anxiety Stress Scale (DASS; Lovibond and Lovibond, 1995), the Impact of Events Scale-Revised (IES-R; Weiss, 2007), the Trauma Symptom Checklist for Children (TSCC; Briere, 1996), the Abuse Attribution Inventory (Feiring et al., 2002), the Rosenberg Self-Esteem Scale (Rosenberg, 1965), and the Event-Related Rumination Inventory (Cann et al., 2011). The interview data were analyzed using thematic analysis (Braun and Clarke, 2006), and the questionnaire data were analyzed using correlational analysis.

Mixed-method studies, such as (Joleby et al. 2021), created a quantitative and qualitative coding manual, based on a set of variables taken from (Ernberg et al. 2018), to examine court transcripts, and used thematic analysis (Braun and Clarke, 2006) and binary logistic regression. Hamilton-Giachritsis et al.'s (2017) mixed-method study used interviews, open-ended survey questions, and a combination of psychometric measures, including the DASS (Lovibond and Lovibond, 1995), the IES-R (Weiss, 2007), and the TSCC (Briere, 1996). They undertook statistical analyses as suitable to the type of data, and used thematic analysis to analyze the qualitative data (Braun and Clarke, 2006). In a further study, (Hamilton-Giachritsis et al. 2021) developed a mixed-methods pilot questionnaire using both cross-tabulation and thematic analysis (Braun and Clarke, 2006) (see Appendix A for an overview of the 18 articles included in the review).

### Quality appraisal

The methodological quality of the included articles was assessed using the most recent version of the Mixed-Methods Appraisal Tool (MMAT; Hong et al., 2018). The MMAT was selected as it provides a single, validated tool for appraising diverse study designs and evaluating key criteria, such as research rationale, sample representativeness, and coherence of data collection and analysis (Hong et al., 2019; Pace et al., 2012). While the MMAT's qualitative items demonstrate lower reliability, this is consistent with the ongoing academic debate surrounding the appraisal of qualitative research, which is regarded as inherently complex due to the diversity of findings, and the role of the researcher throughout the design and analytic process (Pace et al., 2012).

Following MMAT guidance, which discourages the use of a single aggregate score, this review followed Hong et al.'s (2018) directive, and adopted a descriptive star-rating system to summarize each study's quality (i.e., 5^*^ indicates that all the criteria were met). To ensure reliability, a second independent reviewer rated 25% (*n* = 5) of the articles, and any disagreements in ratings were resolved through discussion. This process resulted in 16 studies being rated as 5^*^, and two studies as 4^*^ (see Appendix B for an overview of the MMAT ratings across the included studies).

### Data analysis

A narrative synthesis approach, guided by (Popay et al. 2006), was selected to synthesize the findings from the included articles. Within this framework, and after relevant data were extracted and tabulated, thematic analysis (Braun and Clarke, 2006) was employed to identify patterns of meaning across the datasets. This method was chosen for its theoretical flexibility, which makes it particularly suitable for analyzing data derived from various research paradigms as were present across the included studies.

**Figure 2:**
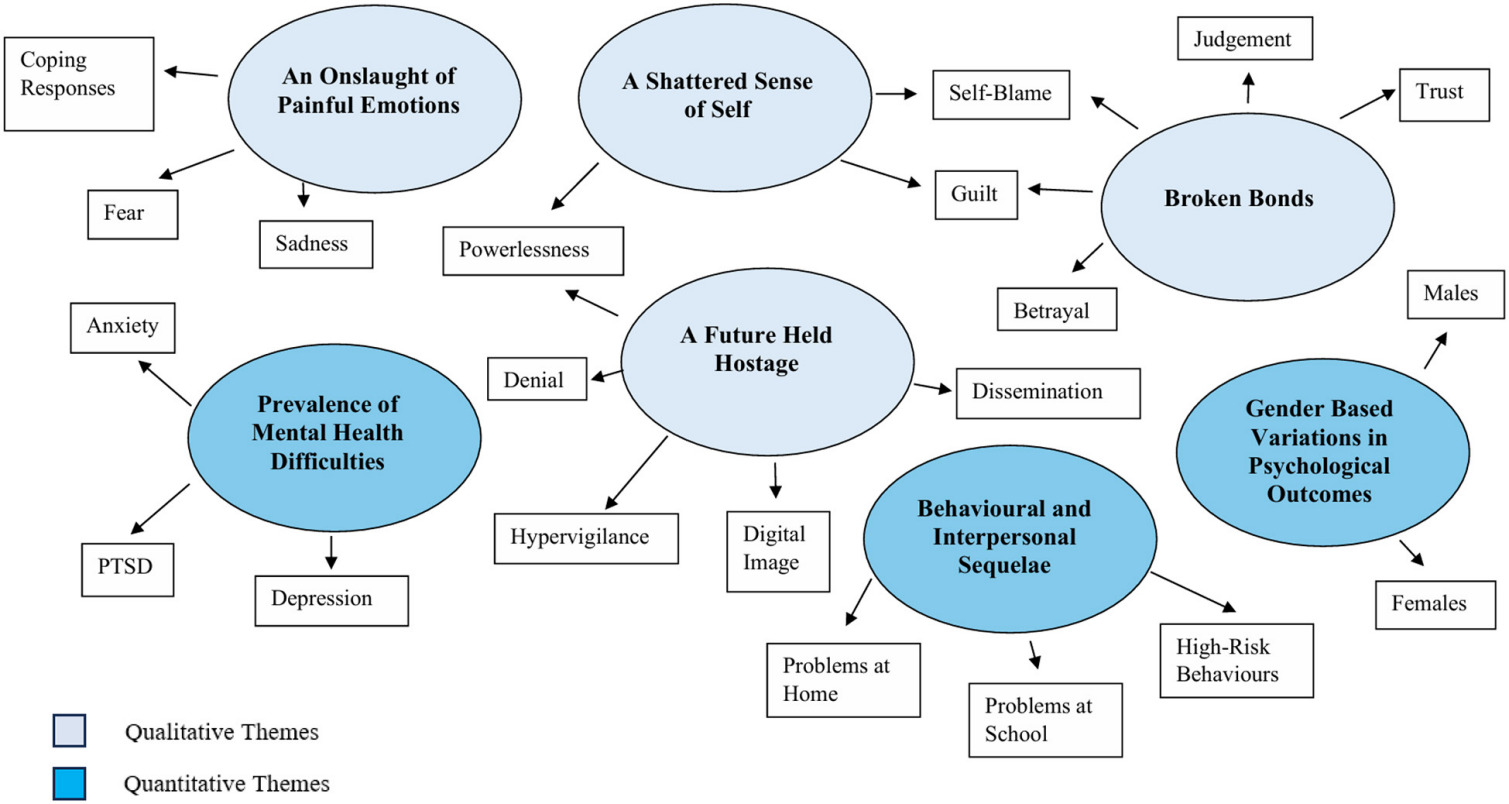
Thematic map.

As proposed by (Popay et al. 2006), findings were organized using tabulation, and the six steps by (Braun and Clarke 2006) were followed thereafter. Step 1 included the researcher familiarizing themselves with the datasets. This involved becoming immersed within the data, and re-reading the included articles. During this initial stage, the researchers noted down any questions or initial observations that arose from the data. Once the researcher became familiarized with the data, they moved on to the second step, which involved reading the data and assigning codes that go beyond the participants' meaning to provide interpretation of the data. Due to the review including articles reporting on studies that had used a range of methodological paradigms, the researcher used descriptive and interpretive codes. Step 3 involved reviewing the codes and searching for themes which represented a patterned response or meaning from the codes. A thematic map was compiled by clustering codes to represent overarching themes within the data (see Figure 2). Once initial themes were identified, the researcher re-read the included articles to ensure that the themes appropriately reflected the coded data.

As part of Step 4, the researcher reviewed all themes in relation to the dataset and extracts. Codes were relocated or discarded to meaningfully capture the essence of the data and improve coherence. Step 5 consisted of defining and naming the themes. The researcher considered whether the themes were relevant in answering the research question, and if they reflected an analytic narrative from the compiled data extracts. Short synopses of each theme were written to summarize the core meaning, and concise descriptive labels were selected to convey a vivid sense of what the theme encompassed (Braun and Clarke, 2006).

### Reflexivity

To ensure rigor and quality, it is important to reflect on the role of the researcher throughout the process of analysis and interpretation. This study sits within an interpretivist paradigm, which asserts that reality is socially constructed and shaped through subjective experience (Sundler et al., 2019). Guided by a phenomenological approach, the researcher aimed to understand the lived experiences of victim-survivors in their own words. To achieve this, thematic analysis was employed to interpret how participants make sense of their experiences, acknowledging that multiple, co-existing realities can be constructed from the same events.

With this in mind, the researcher acknowledges that their own background and experience may impact their interpretation of the findings. Further to this, the researcher has clinical experience of working with victim-survivors of TA-CSA, which may have subconsciously influenced their perception of the data. Throughout analysis and interpretation of the findings, the researcher made a conscious and deliberate effort to attune to their own feelings and reactions to the participants' narratives in the studies. This state of awareness, according to (Berger 2015), allows the researcher to identify the effect of the personal, situational and contextual aspects of themselves in relation to the process of analysis, helping maintain awareness of the similarities and differences within the findings.

## Results

Results from the qualitative and quantitative studies, as well as the qualitative and quantitative components of the mixed-methods studies, will be presented apart, drawing on Hong et al.'s (2020) convergent segregated approach to synthesis and integration of findings.

### Qualitative findings

Four themes were identified that represent the qualitative findings from the five qualitative and four mixed-methods studies (ECPAT International, 2022; Gemara et al., 2022; Hamilton-Giachritsis et al., 2017, 2020, 2021; Joleby et al., 2021; Manrai et al., 2021; Martin, 2014; Whittle et al., 2013): (1) An Onslaught of Painful Emotions, (2) A Shattered Sense of Self: Self Blame and Guilt, (3) A Future Held Hostage: Image Permanence and Hypervigilance, and (4) Broken Bonds: Social Isolation and Relational Rupture.

#### Theme 1: an onslaught of painful emotions

Following experiences of TA-CSA, victim-survivors in six studies described being consumed by overwhelming emotions (ECPAT International, 2022; Gemara et al., 2022; Hamilton-Giachritsis et al., 2017, 2020; Joleby et al., 2021; Whittle et al., 2013). The initial emotional impact of the abuse was often not a single, definable feeling, but a pervasive and confusing mix of sadness, anxiety and stress. One participant stated:

“*I was stressed and cried”* (ECPAT International, 2022, p. 39),

while another described feeling

“*really low and bad about myself.”* (Hamilton-Giachritsis et al., 2020, p. 5)

Emotional responses were frequently described when remembering the abuse:

“*It's so strong, all the feelings, everything that happened returned…whenever you think its gone, it comes back”* (Gemara et al., 2022, p. 6),

and being endured long-term:

“*Even four years later, it still affects me every day.”* (Hamilton-Giachritsis et al., 2017, p. 28).

Participants also reported symptoms associated with PTSD, such as having “*nightmares almost every night,”* which left them feeling drained, exhausted, and fundamentally distressed (Hamilton-Giachritsis et al., 2020, p. 5). The effect on those around victim-survivors was also noted, with one participant describing the abuse constituting “*emotional distress for the whole family,”* suggesting that the psychological impact of TA-CSA may permeate wider social systems (Whittle et al., 2013, p. 62).

A central element compounding this distress was a profound sense of helplessness, often linked directly to the technological nature of the abuse experience. The lack of control over the existence and potential dissemination of abuse images created a specific and enduring form of anxiety:

“*Yes I was worried, I was afraid they would take those photos and upload and share, share them with others to look at.”* (ECPAT International, 2022, p. 34)

This feeling of powerlessness appeared to be linked to a lack of control and victim-survivors feeling

“*Stuck that I couldn't do anything about it”* (ECPAT International, 2022, p. 29).

These feelings were identified as significant barriers to disclosure, with one participant explaining that they simply “*went home and hid the truth”* out of a sense of helplessness, shame and fear. For some, emotional responses also manifested alongside physical expressions of pain, with one participant reportedly “*sobbing”* and making a “*whining sound”* when incited to perform sexual acts on themselves via webcam (Joleby et al., 2021, p. 171).

For many victim-survivors, the distress escalated into life-threatening, maladaptive coping responses. The psychological pain often became so unbearable that suicide was contemplated as the only means of a viable escape: “*the idea of suicide had become the master already”*; “*I felt like committing suicide because I felt so embarrassed*.” While closely related, a distinct manifestation of this distress was the use of self-injurious behavior. The qualitative data revealed that this was not always a suicidal act, but rather a desperate attempt to manage or externalize internal pain:

“*I never wanted, at that point, to end my life, but it was a way…I felt if I was feeling pain, I was still feeling”* (Hamilton-Giachritsis et al., 2017, p. 28).

This also included disordered eating—while some stopped eating entirely:

“*I lost weight because I was very stressed a lot. I was depressed and all”* (ECPAT International, 2022, p. 39),

others over-ate:

“*I would lock myself in and eat in my room alone.”* (ECPAT International, 2022, p. 41)

Beyond the helplessness and maladaptive coping responses, victim-survivors also experienced feelings of anger and confusion:

“*I had aggression issues after all of this happened”* (Whittle et al., 2013, p. 62).

Confusion was reflected in the fear victim-survivors reported, not of their abuser, but of their own personal culpability, stating that they were

“*Afraid that the police would come to arrest me”* (ECPAT International, 2022, p. 27).

This highlights the complex cognitive and emotional dissonance that victim-survivors have to endure. Overall, this theme illustrates that the emotional impact of TA-CSA is not a static list of negative feelings, but a dynamic, complex, and a deeply painful experience that provides the foundation for more enduring psychological consequences to develop.

#### Theme 2: a shattered sense of self: self-blame and guilt

Beyond the initial emotional responses, six studies revealed that TA-CSA inflicts a lasting on victim-survivors' sense of self, shattering their identity through a cycle of self-loathing, guilt and blame (Gemara et al., 2022; Hamilton-Giachritsis et al., 2017, 2020, 2021; Joleby et al., 2021; Whittle et al., 2013). The experience fundamentally altered how victim-survivors perceived themselves, with many describing a painful disconnection from who they were prior to the abuse incident, and having

“*Totally changed”* or being “*a new person”* (Gemara et al., 2022, p. 5).

One participant likened themselves to being less mature prior to the abuse, alluding to the abuse event taking away their childhood innocence:

“*I was too much like a girl then, I behaved differently”* (Gemara et al., 2022, p. 5).

This “transformation” was consistently framed as negative, leaving victim-survivors feeling that their identity had been irrevocably

“*Ruined”* (Hamilton-Giachritsis et al., 2020, p. 5),

and expressing a deep-seated hatred of themselves after the abuse:

“*I hated myself mostly, I didn't like myself at all.”* (Hamilton-Giachritsis et al., 2021, p. 6)

Such visceral feelings of self-loathing, hatred and disgust were commonly noted across studies. Within the analysis of court proceedings, victim-survivors were described to express and experience disgust when evidence of their abuse was reviewed:

“*It is clear from the video that she does not act voluntarily. She shows pain and disgust in connection with several acts and begs him to let her stop.”* (Joleby et al., 2021, p. 170)

This often manifested as a sense of being physically or metaphorically unclean, and feeling

“*dirty”* (Hamilton-Giachritsis et al., 2021, p. 6):

“*I felt disgusted with myself. I ran the shower as hot as I could and I sat in the shower, under the boiling water, hoping that it would make me feel better.”* (Hamilton-Giachritsis et al., 2017, p. 28)

This feeling of “dirtiness” was not only felt internally, but also reinforced by the external world: “*people were calling me a slag, I couldn't get on the bus,”* reinforcing their already existing internal feelings of shame and degradation (Hamilton-Giachritsis et al., 2017, p. 39).

A key psychological mechanism seen to drive this distorted sense of self were pervasive feelings of guilt and self-blame. Victim-survivors consistently questioned their own actions and judgement, holding themselves culpable for the abuse they had endured:

“*Of course I'm going to blame myself because I put myself in lots of these situations”* (Hamilton-Giachritsis et al., 2020, p. 5).

This was further evident in the self-depreciating statements, describing themselves as

“*a stupid idiot that agreed and did whatever she told me to do”* (Gemara et al., 2022, p. 7),

and

“*a little whore”* (ECPAT International, 2022, p. 3).

Other victim-survivors tormented themselves with questions like

“*Why did I not see this coming?”* (ECPAT International, 2022, p. 35),

and feeling

“*like this was all my fault.”* (Hamilton-Giachritsis et al., 2017, p. 34)

Studies indicated that this internalized self-blame was often validated and compounded by the reactions of others. Blame was placed on the victim-survivor by the very people who should have protected them:

“*my dad came and he hit me, yeah, he blamed me for that completely, that it was all my fault, that wasn't the pedophile that groomed me, that was my fault”* (Whittle et al., 2013, p. 63).

This external condemnation was also evident from peers, who would focus on perceived compliance rather than circumstances of manipulation or coercion:

“*Why did you carry on with it when he gave you a choice?”* (Hamilton-Giachritsis et al., 2020, p. 7).

This dynamic, in which victim-survivors were stigmatized by adults who attempted to educate them on ways of how to

“*behave on the internet”* (Hamilton-Giachritsis et al., 2020, p. 7),

led some to feeling like the abuse experience was something they could have avoided:

“*I let it happen, looking back it was so obvious just by the way they were talking…the whole webcam's broken and all this…I think, how did I not notice?”* (Hamilton-Giachritsis et al., 2017, p. 30).

Although professionals working with victim-survivors were able to recognize that

“*Some young people can find it very difficult to see they are not to blame”* (Hamilton-Giachritsis et al., 2021, p. 2),

most often their internalization of guilt was reinforced by external blame, contributing to worsening mental health outcomes, and impeding their ability to recover. Overall, this theme demonstrates that distortions of the self are not a simple consequence of the abuse, but a complex process driven by the interplay of victim-survivors' internalized self-blame, and its external reinforcement of their social environment.

#### Theme 3: a future held hostage: image permanence and hypervigilance

A defining feature of TA-CSA that emerged across the qualitative data within all nine studies was its unique nature, driven by the permanence of the digital material which holds victim-survivors' psychological wellbeing and future prospects hostage (ECPAT International, 2022; Gemara et al., 2022; Hamilton-Giachritsis et al., 2017, 2020, 2021; Joleby et al., 2021; Manrai et al., 2021; Martin, 2014; Whittle et al., 2013). Unlike the majority of CSA within the physical world, where the memory is the primary remnant of the event, TA-CSA most often creates a tangible, distributable artifact of the abuse: it

“*Is no longer silent and hidden…it is now recorded for the world to see. It is permanent and it will never be erased. The abuse is ongoing”* (Martin, 2014, p. 104); “*there is no resolution to the abuse, in that even if material is removed the offender (or someone else) could reupload them at any time.”* (Hamilton-Giachritsis et al., 2021, p. 7)

This sentiment was echoed by victim-survivors' own understanding:

“*You can delete it, but it's never fully deleted.”* (Hamilton-Giachritsis et al., 2020, p. 7)

This sense of permanence fundamentally removed the possibility of closure for victim-survivors, creating what a professional termed as a “*ghost in the closet,”* with the uncertainty of when it may reappear being regarded as

“*Devastating”* (Martin, 2014, p. 105).

Across studies, this lack of resolution was seen to leave victim-survivors in a state of enduring helplessness, consumed by a lack of control over their own violation, and tormented by questions such as

“*Where was my photo?”* (ECPAT International, 2022, p. 35).

Furthermore, they often dealt with image-related blackmail in the form of threats around dissemination to peers or family to guarantee silence. In the study by (Hamilton-Giachritsis et al. 2020), this led to feelings of powerlessness, with victim-survivors reporting that abusers were threatening them:

“*I can use that video, I can put it anywhere I want, send it to whoever I want”* (p. 7); “*I know where you live, I know this and I know that, I'll come and harm your family if you don't do things, and I felt like it's never going to stop.”* (p. 7)

Given the presence of this constant threat, anxiety frequently manifested as severe intrusions and PTSD symptoms. The abuse felt inescapable:

“*I'd be watching telly and I'd literally think, ‘oh I've stopped thinking about it,' and as soon as I think that then, boom it's in my head again”* (Hamilton-Giachritsis et al., 2017, p. 28).

Intrusions were often triggered by everyday sensory information like

“*certain senses or noises or names”* (Hamilton-Giachritsis et al., 2020, p. 5),

and the presence of technology itself, with one participant expressing a deep aversion to being filmed at interview:

“*I hate this camera… I do not like it, and I do not want it to be here”* (Gemara et al., 2022, p. 8).

Faced with this dilemma, victim-survivors developed complex and often contradictory ways of coping. While some reported struggling to stop their recollections, with them

“*Playing over and over, like a film in my mind”* (Hamilton-Giachritsis et al., 2017, p. 28),

others engaged in suppression and/or avoidance:

“*I do not remember any of it. I tried so hard to get it out of my brain and somehow I succeeded”* (Gemara et al., 2022, p. 7); “*Nothing happened to me. I never meet people who I do not know. I would never do such a thing”* (Gemara et al., 2022, p. 5);

“*From now on, I'm going to respond to all of your questions with ‘I don't remember' because for me, all of this is dead. That's it. For me she [the perpetrator] does not exist”* (Gemara et al., 2022, p. 5).

Again others acknowledged what had happened to them, and recognized the likely long-term nature of the impact:

“*I don't think I'm gonna forget this whole thing, yeah it will get distant as I grow older but I don't think I'll ever forget it”* (Whittle et al., 2013, p. 62).

Feelings of hypervigilance to exposure and fears following threats therefore perpetuated an on-going cycle of anxiety and distress for victim-survivors. More specifically, victim-survivors expressed feelings of fear directly related to the threat of re-exposure as having the potential to “*haunt”* them long after the event had ended (Martin, 2014, p. 105), as well as damage their reputation and impact their life negatively:

*In your job application, they can look at your CV, look at your name, just not hire you on the spot when they know you have some icky sexual abuse claim going on and they just don't want to associate with you*. (Manrai et al., 2021, p. 7)

In the study by (Joleby et al. 2021), victim-survivors reported fearing “*that the pictures would reach the public”* (p. 170). The lived reality of a future held hostage stood in stark contrast to the views of some professionals who minimized the harm of TA-CSA as being “*not as serious”* or as “*intrusive”* as sexual abuse in the physical world (Martin, 2014, p. 107). However, this view was challenged by some professionals, and recognized as “*dismissive,”* while at the same time acknowledging that not treating TA-CSA to be as damaging as sexual abuse in the physical world was a societal problem:

“*So that's kind of minimizing, denying of harm. I think, as a society we do that. It's just an image, right?”* (Martin, 2014, p. 107)

Overall, this theme demonstrates that the permanence of images creates a unique form of trauma response, often trapping victim-survivors in cycles of intrusive memory, hypervigilance and fear, and a sense of a future that has been irretrievably compromised.

#### Theme 4: broken bonds: social isolation and relational rupture

A sense of internal damage, brought on by TA-CSA and radiating outwards, was identified across six studies, causing a significant breakdown of interpersonal relationships, and often leading victim-survivors to experience a state of deep social isolation (ECPAT International, 2022; Hamilton-Giachritsis et al., 2017, 2020, 2021; Joleby et al., 2021; Whittle et al., 2013). The psychological impact experienced did not remain contained within the individual, but actively ruptured their social support networks, including their family.

For some, disclosure of abuse brought comfort, being reassured by family that “*it's not your fault,”* which helped to alleviate feelings of blame (Hamilton-Giachritsis et al., 2017, p. 29). In contrast, others described being fundamentally alienated from their parents, unable to “*look at them in the same way,”* and feeling emotionally “*closed off”* and “*awkward”* (Whittle et al., 2013, p. 62), after being “*warned by mum”* about the dangers of talking to strangers online, and/or feeling

“*That my parents did not trust me anymore.”* (Whittle et al., 2013, p. 62)

Feelings of shame and self-blame, as detailed within previous themes, frequently acted as a significant barrier to turning to caregivers for support. This was described by victim-survivors as being due to feelings of having failed their parents (Whittle et al., 2013, p. 63) or causing familial “*embarrassment”* (Hamilton-Giachritsis et al., 2021, p. 7). In some cases, this led to a breakdown in relationships:

“*A worse relationship now than before the event”* (Joleby et al., 2021, p. 174),

With others no longer living at home or being disowned by the wider family who “*chose not to believe”* them upon disclosure (Hamilton-Giachritsis et al., 2021, p. 7).

A breakdown of relationships extended beyond the family and into victim-survivors' peer groups. Participants reported intentionally “*pulling back from relationships,”* feeling emotionally “*distant,”* and unable to connect with friends (ECPAT International, 2022, p. 20/p. 41). This withdrawal was often driven by a form of social hypervigilance:

“*When I'm around people, I always think they're thinking about what happened, like they might know about it”* (Hamilton-Giachritsis et al., 2020, p. 7),

and the fear of judgement made social interactions fraught with anxiety, ultimately causing victim-survivors to retreat into isolation. This was exacerbated when the abuse became known publicly, with participants recalling being ostracized and shamed by their peers, making social spaces feel unsafe (Hamilton-Giachritsis et al., 2017). The psychological mechanism appearing to drive this social collapse was a pervasive breakdown of trust. This manifested as a general mistrust that contaminated all social interactions, with one participant reporting to

“*Not feel safe anywhere”* (Joleby et al., 2021., p. 174),

Feeling “*hostile”* around men, being unable to

“*Give guys a chance to prove themselves as not being horrible people”* (Hamilton-Giachritsis et al., 2020, p. 5),

and generally “*having problems trusting other people, especially men”* (p. 174).

The erosion of trust was further compounded by feelings of institutional betrayal. (Whittle et al. 2013) noted both positive and negative interactions with police, ranging from one participant describing how “*they were brilliant, they were, couldn't ask for anybody better to have come picked me up and to be there with me by my side”* (p. 66), to another stating that “*they just talk to you dead nasty like you've done something wrong”* (p. 67). Other studies included accounts from victim-survivors of teachers who “*didn't really care much,”* and being suspended and placed into isolation within a schooling environment after attempting to disclose:

“*That was the last time I ever spoke to anyone about it, because…they didn't hear me”* (Hamilton-Giachritsis et al., 2017, p. 35).

Alongside associated mental health outcomes, such experiences contributed to some victim-survivors only going to school “*when she is able to,”* while others had to take

“*A leave of absence.”* (Joleby et al., 2021, p. 175)

Ultimately, this systematic dismantling of relational and social bonds culminated in profound isolation for some victim-survivors. One participant described becoming increasingly withdrawn and “*introverted,”* wanting no contact from others and resorting to locking themselves in their bedroom to eat alone (ECPAT International, 2022, p. 41). This retreat from the external world was a direct consequence of the ruptures with family, social alienation from peers, and a deep-seated mistrust of others. Joleby et al.'s (2021) research, on the other hand, reported that while some victim-survivors were seen to withdraw into isolation, others developed a fear of solitude, and described being unable “*to do things on her own,”* or to “*manage to be alone so a family member accompanied her to school every day for four months, until she received a resource person at school”* (p. 175). Whether manifesting as a disconnection from others or a fear of being alone, TA-CSA was shown to fundamentally affect victim-survivors' capacity for safe and healthy relationships, leaving them to frequently navigate the significant psychological impact of their victimization alone.

### Quantitative findings

Three themes were identified that represent the quantitative findings within the nine quantitative and four mixed-methods studies (De Santisteban and Gámez-Guadix, 2018; Dönmez and Soylu, 2019; Gámez-Guadix et al., 2021; Guerra et al., 2021, 2022; Hamilton-Giachritsis et al., 2017, 2020, 2021; Joleby et al., 2021; Mitchell et al., 2001; Say et al., 2015; Wells and Mitchell, 2007; Ybarra et al., 2004), namely: (1) Prevalence of Mental Health Difficulties, (2) Behavioral and Interpersonal Sequelae, and (3) Gender-Based Variations in Psychological Outcomes.

#### Theme 5: prevalence of mental health difficulties

Quantitative data across the nine studies demonstrated that TA-CSA is a significant predictor for the development of a wide range of severe and enduring mental health difficulties and high-risk symptomologies (De Santisteban and Gámez-Guadix, 2018; Dönmez and Soylu, 2019; Gámez-Guadix et al., 2021; Guerra et al., 2022; Hamilton-Giachritsis et al., 2017, 2020; Mitchell et al., 2001; Say et al., 2015; Wells and Mitchell, 2007). The evidence points to an alarming picture of adverse mental health outcomes for victim-survivors, with impacts moving beyond subjective distress to quantifiable clinical outcomes. The scale of these adverse outcomes was illustrated in the study by (Wells and Mitchell 2007) who reported that 68% of children with experiences of TA-CSA met the criteria for a lifelong DSM-IV diagnosis. Moreover, the same study identified that mood disorders, including depression and bipolar, were particularly prevalent in samples of victim-survivors, with 54% having a lifetime diagnosis, and the likelihood of comorbidity being significantly higher in cases of TA-CSA due its complex, multi-faceted nature.

Among the various conditions examined, depression was the most consistently reported and statistically significant outcome. Multiple studies identified a significant link between experiencing TA-CSA and increased levels of depressive symptomology (De Santisteban and Gámez-Guadix, 2018; Dönmez and Soylu, 2019; Gámez-Guadix et al., 2021; Guerra et al., 2022; Say et al., 2015). These findings were supported by the data from the mixed-method studies, using validated psychometric tools. For example, using the Depression Anxiety Stress Scale, (Hamilton-Giachritsis et al. 2020) found that 54% of their sample reached clinically relevant levels for depression. An earlier study, by the same group of researchers, found that out of 30 participants, 18 obtained scores indicating severe levels of depressive symptoms (Hamilton-Giachritsis et al., 2017). Similarly, anxiety disorders were a frequent outcome, with one study reporting a 24% lifetime prevalence rate (Wells and Mitchell, 2007), and another reporting this presentation to incrase following TA-CSA for 62% of the sample (Hamilton-Giachritsis et al., 2020). A high correlation between the two was further found in the study by (Gámez-Guadix et al. 2021), who attributed this to the significant levels of coercion and manipulation inherent in TA-CSA.

Due to the traumatic nature of TA-CSA, PTSD was identified as a common and enduring consequence (Dönmez and Soylu, 2019; Hamilton-Giachritsis et al., 2017; Mitchell et al., 2001; Say et al., 2015; Wells and Mitchell, 2007). (Wells and Mitchell 2007) reported that a lifetime PTSD diagnosis was nearly five times more likely in cases of TA-CSA compared to other problematic internet experiences (19% vs. 4%, *p* < 0.001). This was further supported by (Dönmez and Soylu 2019) who reported the rate of developing PTSD following TA-CSA to be 57% in their sample. In the study by (Hamilton-Giachritsis et al. 2017), four out of five participants received a score of clinical concern, as assessed by the Impact of Events Scale Revised (Hamilton-Giachritsis et al., 2017).

Across the studies, high rates of behavioral symptoms often associated with mental health difficulties, specifically self-injurious behavior and suicidality, were revealed. In the study by (Hamilton-Giachritsis et al. 2017), 15 (out of 16) participants cut themselves, seven deprived themselves of food, and one in seven attempted suicide. Similarly, using the Trauma Symptom Checklist for Children (Briere, 1996), (Hamilton-Giachritsis et al. 2020) reported that 54% of children presented with suicidal thoughts, and nearly 10% attempted suicide. Overall, these findings provide alarming quantifiable evidence of the severe and enduring psychological impact following TA-CSA, and suggest that it is a potent driver of a wide range of significant and high-risk symptomologies suffered by victim-survivors.

#### Theme 6: behavioral and interpersonal sequelae

Beyond the prevalence of adverse mental health outcomes, the quantitative data across seven studies revealed that TA-CSA is strongly associated with a range of externalizing behaviors and disruptions to interpersonal functioning, affecting victim-survivors across multiple life domains (Dönmez and Soylu, 2019; Gámez-Guadix et al., 2021; Guerra et al., 2022; Hamilton-Giachritsis et al., 2017; Joleby et al., 2021; Wells and Mitchell, 2007; Ybarra et al., 2004). This supports findings that demonstrate that internalized psychological harm radiates outward, tangibly damaging social bonds and developmental trajectories of victim-survivors. For example, (Wells and Mitchell 2007) reported that young people with a history of TA-CSA experienced conflict with caregivers (76%), and disciplinary problems at home (40%). This frequently escalated into more extreme behaviors, with young people being three times more likely to abscond from home (compared to a control group with other problematic internet experiences; 22% vs. 7%, *p* = 0.002; Wells and Mitchell, 2007).

Interpersonal disruption often extended beyond the sphere of the family and into institutional settings and peer relationships. A third of victim-survivors (33%) reported difficulties in sustaining interpersonal relationships, suggesting an impact in their ability to form and maintain healthy connections with their peers (Wells and Mitchell, 2007). This difficulty in interpersonal functioning was often mirrored in a decline in academic performance and behavior within educational settings, namely disciplinary problems (30%), and failing grades (34%), suggesting a direct association between the impact of TA-CSA and victim-survivors' ability to engage successfully therein. These findings were supported by (Hamilton-Giachritsis et al. 2017), who further reported high rates of sleep disturbances that arguably impacted on victim-survivors' ability to engage with and attend school.

Three of the studies suggested that victim-survivors were at an increased risk of presenting with antisocial behaviors and/or developing personality disorders (Dönmez and Soylu, 2019). More specifically, (Guerra et al. 2022) reported higher rates of displaying antisocial and/or aggressive behaviors, and (Wells and Mitchell 2007) reported a notable prevalence of diagnosed conduct disorders (10% current, 14% lifetime). Victim-survivors were further found to engage in high-risk coping strategies at an increased rate, including drug and alcohol use (26%), and risky sexual behaviors (48%) (all comparisons were made with a control group and/or counterparts with no abuse experiences). We would argue that in attempting to process their abuse, victim-survivors may exhibit trauma responses that manifest as harmful and/or problematic behaviors, which inadvertently prolong their cycle of distress.

The quantitative data further revealed that psychological and behavioral consequences of TA-CSA frequently created a cycle of vulnerability and re-victimization. According to (Ybarra et al. 2004), children who had experienced TA-CSA presented with learned helplessness that contributed to lower assertiveness, and thereby placed them at increased risk of re-victimization. Similarly, (Gámez-Guadix et al. 2021) suggest that poor psychological adjustment creates vulnerabilities for repeat abuse experiences, further worsening mental health and behavioral outcomes. (Wells and Mitchell 2007), in fact, found that victim-survivors of TA-CSA were more than three times more likely to experience repeat sexual victimization in their lifetime when compared to a control group (56% vs. 17%, *p* < 0.001). Furthermore, in the study by (Hamilton-Giachritsis et al. 2017), 10 (out of 26) participants reported experiencing unwanted sexual contact/encounters following TA-CSA, and 30% of children in Joleby et al.'s (2021) sample talked about how loneliness had made them more vulnerable to speaking to someone online, and thereby increased their risk of re-victimization. Overall, this theme demonstrates that the range of behavioral and interpersonal consequences not only disrupt victim-survivors' ability to cope, and engage socially with others, but also place them at an increased risk of experiencing harm through future instances of re-victimization.

#### Theme 7: gender-based variations in psychological outcomes

While TA-CSA is profoundly damaging to all young people, the quantitative data suggest that gender can significantly shape the nature of mental health and behavioral outcomes, although findings are at times contradictory. For example, (Guerra et al. 2021) found females to be experiencing significantly higher levels of depressive symptomatology than males (*p* < 0.01). This was further supported by (Ybarra et al. 2004), with females being 60% more likely than males to report depressive symptoms following TA-CSA. (Guerra et al. 2021) argue that the offender's gender was a critical factor for both male and female victim-survivors, with abuse by a male adult being associated with the highest levels of subsequent experiences of depression. Beyond depression, female victim-survivors showed higher rates of suicidality (27%) when compared to male victim-survivors (10%) (Wells and Mitchell, 2007), and extremely low levels of self-esteem, with the average falling into the lowest 5% of female adolescents in the UK (Hamilton-Giachritsis et al., 2021).

On the contrary, the qualitative data frequently pointed toward a higher prevalence of externalizing behaviors among male victim-survivors. In the study by (Wells and Mitchell 2007), males were significantly more likely than their female counterparts to engage in high-risk and antisocial acts post-victimization, including bullying (19% vs. 6%), running away from home (35% vs. 22%), using substances (26% vs. 14%), engaging in harmful and/or problematic sexual behaviors toward other children (16% vs. 2%), and encountering disciplinary problems at school (45% vs. 35%). The same study found that males had higher rates of comorbid pre-diagnosed mental health difficulties (32% vs. 8%), and reported higher overall rates of anxiety than females (53% vs. 45%), painting a complex picture of both internal distress and external coping behaviors. Overall, the findings reported across the included studies suggest an interplay of abuse experience-related factors that have as their consequence a number of mental health and behavioral outcomes, with females being more likely to internalize their distress, and males being more likely to externalize their distress. The presence of contradictory findings suggests that gender is one of several intersecting variables that requires further investigation to fully understand the psychological sequelae of TA-CSA for female and male victim-survivors.

## Discussion

The aim of the present review was to explore the psychological impact on and mental health outcomes for victim-survivors who have experienced TA-CSA. While existing literature provides important insights into its prevalence and risk factors, a significant gap remains in understanding victim-survivors' lived experiences, and specifically in terms of the psychological impact on and mental health outcomes for them. This review addressed this by systematically synthesizing studies that adopted qualitative, quantitative and mixed-methods designs to offer an integrated overview of current knowledge and understanding of this phenomenon.

### Summary of findings

The review's findings substantiate the argument that TA-CSA is a distinct phenomenon with unique and potent mechanisms that lead to long-term suffering (Joleby et al., 2020), and demonstrates that features inherent to the digital environment do not merely cause different impacts and outcomes for victim-survivors, but actively perpetuate the psychological harm they endure. A primary contribution of this review is its consolidation of evidence that directly refutes the outdated and harmful misconception that TA-CSA is less impactful than CSA in the physical world (Finch et al., 2020). In fact, our findings highlight that TA-CSA is a complex and psychologically damaging experience (Finkelhor et al., 2023). The studies reviewed align with the wider literature, showing that victim-survivors are frequently subjected to multiple, overlapping forms of abuse, including grooming, coercion to produce abuse material, and its subsequent retrieval and dissemination (Chauviré-Geib and Fegert, 2021). Such multi-faceted abuse, often comprising more than one offender, compounded victim-survivors' trauma responses, and stands in contrast to a singular event. Furthermore, the high rates of individual psychological impact and various mental health outcomes that were revealed, alongside significant comorbidity, further increased victim-survivors' vulnerability to and likelihood of re-victimization.

The presence and permanence of abuse material appeared to function as a unique trauma mechanism, and fundamentally distinguishes experiences of TA-CSA and CSA in the physical world. As noted by (Martin 2014), the creation of tangible and distributable abuse material prevents victim-survivors from ever experiencing a definitive ending, and finding closure, leading to a prolonged cycle of anxiety, threat, and re-victimization. For example, the theme “A Future Held Hostage” provides a rich experiential account of how this mechanism operates. In line with findings by (Gewirtz-Meydan et al. 2019), victim-survivors described an enduring sense of helplessness, lack of control, and being tormented by the knowledge that depictions of their abuse existed somewhere beyond their reach. Here, (Insoll et al. 2022) argue that this lack of control and resolution manifests in a state of hypervigilance, trapping victim-survivors in a perpetual cycle of rumination around it being a matter of time when people may come across their abuse imagery. Both (Ali and Paash 2022) and (Martin 2014) report that victim-survivors experience considerable distress over the abuse material impacting future relationships and educational and/or employment opportunities.

This chronic state of hypervigilance, driven by an unpredictable external threat, is a reflection of the high rates of PTSD reported across the studies included in this review. Unlike trauma surrounding a past event that may be processed over time, the ever-present nature of abuse material represents a permanent threat that appears to repeatedly activate victim-survivors' trauma responses. According to (Insoll et al. 2022), each dissemination constitutes a new violation, and thereby prevents the experience from being processed. This inability to achieve psychological closure was evident in the life-long diagnoses of mental health difficulties. As such, the documentation of abuse experiences is therefore not merely an incidental feature thereof, but constitutes a core aspect in contributing to severe and long-term psychological suffering.

Further to the above, our findings also shed light on the impact TA-CSA has on victim-survivors' relationships and support systems, significantly contributing to their distress, and ultimately increasing their vulnerability to and likelihood of future victimization. For example, the theme “Broken Bonds” powerfully illustrates this concept, and supports findings by (Joleby et al. 2020) in terms of the impact TA-CSA had on victim-survivors' ability to form healthy interpersonal relationships. Victim-survivors further experienced alienation from peers, and ruptures within the family as a result of punitive retribution following disclosures. Interactions with professionals across education and law enforcement often left victim-survivors feeling misunderstood, and poorly to not supported. Such failures by the structures and systems in place to support victim-survivors contextualizes the complexity of their ways of coping, as captured within the theme “Behavioral and Interpersonal Sequelae.” We would argue that social withdrawal, substance misuse, running away from home, and aggressive behaviors, are not solely symptoms of distress, but manifestations of betrayal as a direct consequence of the relational breakdown experienced by victim-survivors, leaving them to cope with the aftermath of TA-CSA alone.

Woven throughout victim-survivors' accounts of post-TA-CSA experiences was the presence of intense feelings of shame, self-blame, guilt, and being at fault, partly facilitated by others' responses to disclosures, but also the effect of grooming, and its inherent blurring of boundaries between exploitation and what may have appeared to be a consensual relationship, which often characterizes TA-CSA (Stänicke et al., 2024). For example, the theme “A Shattered Sense of Self” captured both these feelings, and the sense of “dirtiness” some victim-survivors reported experiencing, while holding themselves culpable for the abuse they suffered. Through grooming, offenders manipulated and exploited victim-survivors' sense of agency, and thereby instilled in them feelings of complicity and culpability (e.g., for having turned on the webcam or sent an image). According to (Mandau 2021), this betrayal, alongside the shame, self-blame, guilt, and feeling at fault, was found to significantly alter victim-survivors' self-concept.

Overall, the findings presented here, and synthesized from the included studies, may be interpreted through the lens of Herman's (1992) theory of complex trauma, which posits that prolonged victimization results in distinct forms of psychological harm, and the development of complex trauma respectively. The first domain of Herman's framework aligns with the themes “An Onslaught of Painful Emotions” and “The Prevalence of Mental Health Difficulties,” representing difficulties in emotional regulation, maladaptive coping strategies, such as self-harming behaviors and suicidality, and high rates of psychopathology. A second domain, referring to changes in relationships, aligns with the theme “Broken Bonds,” which represents experiences of being let down by others. The inherent sense of betrayal and mistrust subsequently contributes to victim-survivors' inability to feel safe in the company of others. A third domain, referring to an altered sense of identity, aligns with the theme “A Shattered Sense of Self.” representing the link between victim-survivors' experiences around shame, self-blame, guilt, and being at fault, and the sense of a loss of self and transformation of identity.

### Clinical implications

The findings from the present review directly challenge existing misconceptions that TA-CSA is less impactful than CSA in the physical world. In fact, it provides an overview of and sheds light on the sheer psychological impact and mental health outcomes suffered by victim-survivors, who have been shown to experience complex and enduring forms of harm, facilitated by the online environment and digital technologies. This has important implications, especially for practitioners and professional services whose work supports victim-survivors, namely to (i) recognize TA-CSA as a form of sexual abuse, and (ii) consider that complex trauma may offer a better conceptualization of their experiences. Approaches to treatment and therapeutic interventions should take into account the unique mechanisms of harm that have been identified, especially around the ever-present nature of abuse material, making closure and recovery an often long and challenging process for victim-survivors.

### Limitations and directions for future research

A number of limitations are important to bear in mind when taking note of the findings presented in this review. Firstly, while most of the included studies reported on the demographics of their participants, information on ethnicity was often absent or missing. Secondly, the sample sizes across most of the included studies were relatively small. While this is not uncommon in light of the topic area, it is worth acknowledging that the findings therefore likely reflect the experiences of victim-survivors who have felt able to come forward, talk about their experiences, and participate in research. In terms of directions for future research, the existing literature would benefit from (i) the representation of more diverse samples by focusing on the psychological impact on and the mental health outcomes for children and young people from different ethnic (minority) groups; and (ii) adopting a more longitudinal perspective, focused on victim-survivors' experiences of recovery over time, including what may help reduce shame, and build a more positive sense of self.

## Data Availability

The raw data supporting the conclusions of this article will be made available by the authors, without undue reservation.
