# Supplementary material for: The psychological impact on and mental health outcomes for victim-survivors of technology-assisted child sexual abuse: a systematic literature review
Source: Front Psychol. 2026 Apr 10;17:1682155. doi: 10.3389/fpsyg.2026.1682155 (PMC13105988; doi:10.3389/fpsyg.2026.1682155)
Supplement: Supplementary file 1 [file Supplementary_File_1.docx]

**Appendices**

**Appendix A.** Overview of Studies

| **Author** | **Country** | **Population Description** | **Primary Aim** | **Paradigm Model** | **Data Collection Method** | **Summary of Results** |
| --- | --- | --- | --- | --- | --- | --- |
| De Santisteban and Gámez-Gaudix (2018) | Spain | 1924 aged 12-14 years old (*M=13.11, SD = 0.79*, 52.9% female, 46.1% male 1.1% not reported) | To analyse the longitudinal relationships between online child sexual victimisation and depression completing measures at T1 and T1, 1 year apart. | Quantitative | Questionnaires | Higher levels of depressive symptoms at T1 predicted an increased likelihood of online sexual solicitation and interaction with adults at T2. In contrast, online sexual solicitation and interaction at T1 did not predict an increase in depression at T2. These findings held true for girls and boys. |
| Dönmez and Soylu (2019) | Canada | 189 young people aged 12-16 years old. | To examine the sociodemographic risk factors of TA-CSA and the association between TA-CSA and psychiatric disorders in a clinical sample. | Quantitative | Questionnaires and interviews | Depressive disorder, borderline personality disorder, and secondary psychiatric diagnosis were significantly higher in adolescents who were exposed to TA-CSA. The rate of PTSD development after exposure to TA-CSA was determined as 57.8%. |
| ECPAT (2022) | Africa, Malaysia and Cambodia | 33 young people aged 16-24 who had experienced TA-CSA when they were under the age of 18. | To explore the psychological consequences for children subjected to online child sexual exploitation and abuse and professionals understanding of the risks associated with online child sexual abuse. | Qualitative | Unstructured Interviews | The study revealed that victim-blaming was a significant inhibitor to disclosure for young people who had experienced TA-CSA. TA-CSA was seen as inherently responsible for a wide range of trauma symptoms and adverse outcomes. |
| Gámez-Guadix et al. (2021) | Spain | 1704 participants aged 15 years and younger (*M = 13.77, SD = 1.13*). 864 participants were female, 828 male. | To develop a new instrument for measuring online grooming. To use this tool to examine the prevalence of different grooming strategies and identify associations with specific mental health outcomes. | Quantitative | Questionnaire | Results identified a significant relationship between different grooming strategies and grooming outcomes in relation to depression and anxiety symptoms. |
| Gemara et al. (2022) | Israel | 20 female children aged 11-13 years (*M=12.25*). | To explore young people’s experiences of TA-CSA during forensic interviews. | Qualitative | Semi- Structured Interviews | Findings revealed children experienced difficulties when confronted with materials related to TA-CSA. They discussed challenges to their wellbeing and had difficulties talking about the abuse and being videotaped by the interviewer. |
| Guerra et al. (2021) | Chile | 18,872 students aged 12-17 (*M = 14.54, SD = 1.42*). 50.8% of participants were female, 49.2% male. | To determine the prevalence of TA-CSA by offender type and compare the associated levels of depression across these groups. | Quantitative | Secondary analysis of survey data. | The gender and age of the abuser were significantly related to depressive symptoms. Higher levels of depression were associated with harassment by an adult male or an offender of unknown age or gender. |
| Guerra et al. (2022) | Chile | 380 participants aged 15–17 years (*M = 16.12, SD = .59*, 49.7% girls, 50.3% boys). | To explore how experiences of TA-CSA relate to internalising and externalising symptoms in adolescents, and to assess the role of perceived peer support in this relationship. | Quantitative | Questionnaires | Results indicated a significant association between experiencing TA-CSA and adverse outcomes, including depression, self-harm and anti-social behaviour. |
| Hamilton-Giachritsis et al. (2017) | UK | 36 young people aged 15-19 years and 52 professionals who worked in the field of sexual abuse. | To explore professional perceptions of TA-CSA and its impact and to develop an understanding of how young people perceive themselves to be impacted by abuse. | Mixed-Methods | Questionnaires, psychometric measures and Interviews | TA-CSA is perceived as having less impact than abuse occurring in the physical world by professionals. Children are often blamed or seen as participating in abuse when it happens online. |
| Hamilton-Giachritsis et al. (2020) | UK | 260 young people aged 19-21 who had experienced TA-CSA when they were under 18. | To explore the impact of TA-CSA from the perspectives of youth, including any complexities that arise from any digital element. | Mixed-Methods | Interviews, questionnaires and screening data. | TA-CSA outcomes appear to be the same as CSA, although viewed as less serious. TA-CSA also has additional complexities that require acknowledgement within guidelines and policies. |
| Hamilton-Giachritsis et al. (2021) | UK | 52 professionals who had worked with children who had experienced child sexual abuse. | To understand how professionals who work with victim-survivors perceive TA-CSA and explore their organisational responses to disclosures of abuse. | Mixed-Methods | Questionnaires and online surveys | Professionals demonstrated a limited understanding of TA-CSA and its impacts, perceiving it to be less impactful than contact CSA. |
| Joelby et al. (2021) | Sweden | Children ages 7-17 years, *(M = 12.3, SD = 1.92*). | To explore how experiences of psychological health were described for children within written court verdicts. | Mixed-Methods | Secondary data -transcripts. | TA-CSA was identified as having a range of psychological consequences. TA-CSA was perceived as threatening, distressing and painful. |
| Manrai et al. (2021) | Scotland and Chile | 51 adolescents aged 15-20 years who were under the age of 18 at the time of abuse. 6 participants from Scotland and 45 from Chile. | To explore what factors facilitate or prevent disclosures of TA-CSA. | Qualitative | Interviews and Focus Groups | Findings attributed lack of knowledge regarding TA-CSA to inhibit disclosure, alongside vulnerability and gender factors. |
| Martin (2014) | Canada | 14 practitioners who had worked with youth up to age 18 who had experienced TA-CSA. | To examine practitioners’ views and experiences working with TA-CSA victim-survivors. | Qualitative | Semi-structured Interviews | Practitioners experienced difficulty conceptualising what constituted TA-CSA and held carrying degrees of concern regarding the effects and impacts. |
| Mitchell, Finkelhor and Wolak (2001) | United States | 1501 youth aged 10-13 (558) and 14-17 (942). | To assess the risk factors associated with TA-CSA and distress. | Quantitative | Telephone Surveys | Overall, 25% of youth reported high levels of distress after TA-CSA. Younger youth more commonly reported levels of distress (P=.005), alongside youth who received aggressive solicitations (P=.001) and youth who used a computer not located within their home (P=.001). |
| Say et al. (2015) | Turkey | 93 youth aged 4-18 years old (*M=14.12*). 84% were female, 16% were male. 74% attended school, 23% had dropped out of school and 3% were preschoolers. Developmental delay was present in 11.8% of participants. | To examine the prevalence of psychiatric correlates of TA-CSA in a sample of child and adolescent victim-survivors. | Quantitative | Interviews, psychiatric diagnosis, cognitive tests. | TA-CSA showed significant association with both penetrative and recurrent forms of sexual abuse. Victims experienced an increased risk for psychiatric disorders, including any psychopathology (4.21 times more likely), depression (3.77 times more likely) and PTSD (2.14 times more likely). |
| Wells and Mitchell (2007) | Turkey | 512 professionals who had worked with children aged 13-17 years who had experienced problematic internet experiences. | To examine demographics, DSM-IV diagnoses and gender differences among youth online sexual exploitation victims. | Quantitative | Surveys | 68% of children who had experienced TA-CSA had a lifetime DSM-IV diagnosis. Clinicians should work closely with authorities to provide comprehensive treatment. |
| Whittle, Hamilton-Giachritsis & Beech (2013) | UK | 6 girls and 2 boys aged 13-18 years (*M = 15.88, SD = 2.17*) who experienced TA-CSA between the ages of 12-14 years (*M = 12.88, SD = 0.84*). | To examine children’s experiences regarding the impact of TA-CSA and experiences of professional involvement post abuse. | Qualitative | Semi-Structured Interviews | Children experiencing long-term risk factors and vulnerability to TA-CSA suffered greater negative impact after abuse compared to those who had protection. |
| Ybarra et al. (2004) | United Kingdom (UK) | 1501 youth aged 10-13 (558) and 14-17 (942). | To examine the relationship between depressive symptomology and TA-CSA. | Quantitative | Secondary analysis of telephone surveys | Adolescents with high depressive symptoms who reported an TA-CSA were more than twice as likely to report emotional distress compared to those with mild or no symptoms (OR= 2.27, 95% CI, 1.03, 2.02) |

**Appendix B.** Overview of Quality Appraisal using the MMAT (Hong et al., 2019)

| **Study Design and Articles** | **Assessment Criteria - MMAT** | | | | | | | |
| --- | --- | --- | --- | --- | --- | --- | --- | --- |
| **Screening Questions (SQ)**  **All Methods** | SQ 1 | Are there clear research questions? | | | | | | |
|  | SQ 2 | Do the collected data allow to address the research questions? | | | | | | |
| **Qualitative** | **SQ**  **1** | **SQ 2** | **Is the qualitative approach appropriate to answer the research question?** | **Are the qualitative data collection methods adequate to address the research question?** | **Are the findings adequately derived from the data?** | **Is the interpretation of results sufficiently substantiated by data?** | **Is there coherence between qualitative data sources, collection, analysis and interpretation?** | **Quality rating** |
| Gemara et al. (2022) | Y | Y | Y | Y | Y | Y | Y | 5* |
| Manrai et al. (2021) | Y | Y | Y | Y | Y | Y | Y | 5* |
| Whittle et al. (2013) | Y | Y | Y | Y | Y | Y | Y | 5* |
| Martin (2014) | Y | Y | Y | Y | Y | Y | Y | 5* |
| ECPAT (2022) | Y | Y | Y | Y | Y | Y | Y | 5* |
| **Quantitative Descriptive** | **SQ**  **1** | **SQ 2** | **Is the sampling strategy relevant to address the research question?** | **Is the sample representative of the target population?** | **Are the measurements appropriate?** | **Is the risk of non-response bias low?** | **Is the statistical analysis appropriate to answer the research question?** | **Quality rating** |
| Dönmez and Soylu (2019) | Y | Y | Y | Y | Y | Y | Y | 5* |
| Gámez-Guadix et al. (2021) | Y | Y | Y | Y | Y | Y | Y | 5* |
| Guerra et al. (2022) | Y | Y | Y | Y | Y | Y | Y | 5* |
| Guerra et al. (2021) | Y | Y | Y | Y | Y | Y | Y | 5* |
| Say et al. (2015) | Y | Y | Y | Y | Y | Y | Y | 5* |
| Ybarra et al. (2004) | Y | Y | Y | Y | Y | Y | Y | 5* |
| **Quantitative**  **Non – randomised studies** | **SQ**  **1** | **SQ**  **2** | **Are the participants representative of the target population?** | **Are measurements appropriate regarding both the outcome and intervention (or exposure)?** | **Are there complete outcome data?** | **Are the confounders accounted for in the design and analysis?** | **During the study period, is the intervention administered (or exposure occurred as intended?** | **Quality Rating** |
| De Santisteban and Gámez-Gaudix (2018) | Y | Y | Y | Y | Y | Y | Y | 5* |
| Wells and Mitchell (2007) | Y | Y | Y | Y | Y | CT | Y | 4* |
| Mitchell et al. (2001) | Y | Y | Y | Y | Y | CT | Y | 4* |
| **Mixed methods studies** | **SQ1** | **SQ2** | **Is there an adequate rationale for using a mixed methods design to address the research question?** | **Are the different components of the study effectively integrated to answer the research question?** | **Are the outputs of the integration of qualitative and quantitative components adequately interpreted?** | **Are divergences and inconsistencies between quantitative and qualitative results adequately addressed?** | **Do the different components of the study adhere to the quality criteria of each tradition of the methods involved?** | **Quality rating** |
| Hamilton-Giachritsis et al. (2020) | Y | Y | Y | Y | Y | Y | Y | 5* |
| Joelby et al. (2021) | Y | Y | Y | Y | Y | Y | Y | 5* |
| Hamilton-Giachritsis et al. (2017) | Y | Y | Y | Y | Y | Y | Y | 5* |
| Hamilton-Giachritsis et al. (2021) | Y | Y | Y | Y | Y | Y | Y | 5* |
